# Cancer risks in Lynch syndrome, Lynch-like syndrome, and familial colorectal cancer type X: a prospective cohort study

**DOI:** 10.1186/s12885-020-06926-x

**Published:** 2020-05-24

**Authors:** Karolin Bucksch, Silke Zachariae, Stefan Aretz, Reinhard Büttner, Elke Holinski-Feder, Stefanie Holzapfel, Robert Hüneburg, Matthias Kloor, Magnus von Knebel Doeberitz, Monika Morak, Gabriela Möslein, Jacob Nattermann, Claudia Perne, Nils Rahner, Wolff Schmiegel, Karsten Schulmann, Verena Steinke-Lange, Christian P. Strassburg, Deepak B. Vangala, Jürgen Weitz, Markus Loeffler, Christoph Engel, on behalf of the German Consortium for Familial Intestinal Cancer

**Affiliations:** 1 Institute for Medical Informatics, Statistics and Epidemiology (IMISE), University of Leipzig, Leipzig, Germany; 2 Institute of Human Genetics, University of Bonn, Bonn, Germany; 3 National Center for Hereditary Tumor Syndromes, University Hospital Bonn, Bonn, Germany; 4 Institute of Pathology, University of Cologne, Cologne, Germany; 5 Medizinische Klinik und Poliklinik IV, Campus Innenstadt, Klinikum der Universität München, Munich, Germany; 6 Center of Medical Genetics, Munich, Germany; 7 Department of Internal Medicine I, University Hospital Bonn, Bonn, Germany; 8 Department of Applied Tumour Biology, Institute of Pathology, University Hospital Heidelberg, Heidelberg, Germany; 9 Cooperation Unit Applied Tumour Biology, German Cancer Research Center (DKFZ), Heidelberg, Germany; 10 Center for Hereditary Tumors, HELIOS Klinikum Wuppertal, University Witten-Herdecke, Wuppertal, Germany; 11 Institute of Human Genetics, Medical Faculty, Heinrich-Heine-University, Düsseldorf, Germany; 12 Department of Medicine, Knappschaftskrankenhaus, Ruhr-University Bochum, Bochum, Germany; 13 Department of Hematology and Oncology, Klinikum Hochsauerland, Meschede, Germany; 14 Medical Practice for Hematology and Oncology, MVZ Arnsberg, Arnsberg, Germany; 15 Department of Surgery, Technische Universität Dresden, Dresden, Germany

**Keywords:** Lynch syndrome, Lynch-like syndrome, Familial colorectal cancer type X, Cancer risk, Prospective surveillance study

## Abstract

**Background:**

Individuals with pathogenic germline variants in DNA mismatch repair (MMR) genes are at increased risk of developing colorectal, endometrial and other cancers (Lynch syndrome, LS). While previous studies have extensively described cancer risks in LS, cancer risks in individuals from families without detectable MMR gene defects despite MMR deficiency (Lynch-like syndrome, LLS), and in individuals from families fulfilling the Amsterdam-II criteria without any signs of MMR deficiency (familial colorectal cancer type X, FCCX) are less well studied. The aim of this prospective study was to characterise the risk for different cancer types in LS, LLS, and FCCX, and to compare these with the cancer risks in the general population.

**Methods:**

Data was taken from the registry of the German Consortium for Familial Intestinal Cancer, where individuals were followed up prospectively within the framework of an intensified surveillance programme at recommended annual examination intervals. A total of 1120 LS, 594 LLS, and 116 FCCX individuals were analysed. From this total sample, eight different cohorts were defined, in which age-dependent cumulative risks and standardised incidence ratios were calculated regarding the first incident occurrence of any, colorectal, stomach, small bowel, urothelial, female breast, ovarian, and endometrial cancer, separately for LS, LLS, and FCCX.

**Results:**

The number of individuals at risk for first incident cancer ranged from 322 to 1102 in LS, 120 to 586 in LLS, and 40 to 116 in FCCX, depending on the cancer type of interest. For most cancer types, higher risks were observed in LS compared to LLS, FCCX, and the general population. Risks for any, colorectal, stomach, urothelial, and endometrial cancer were significantly higher in LLS compared to the general population. No significantly increased risks could be detected in FCCX compared to LLS patients, and the general population. Colorectal and endometrial cancer risks tended to be higher in LLS than in FCCX.

**Conclusions:**

The characterisation of cancer risks in patients with LLS and FCCX is important to develop appropriate surveillance programmes for these specific intermediate risk groups. Larger prospective studies are needed to obtain more precise risk estimates.

## Background

Lynch syndrome (LS) is an autosomal dominantly inherited disorder, which is caused by pathogenic germline variants in one of the DNA mismatch repair (MMR) genes *MLH1*, *MSH2*, *MSH6*, *PMS2* or in the *EPCAM* gene [1–3]. It is estimated that one of 279 individuals in the general population carries a pathogenic MMR gene variant [4]. LS is the most common hereditary colorectal cancer syndrome and is responsible for about 2 to 4% of all colorectal cancers [5–7]. Individuals with LS are at an increased risk of developing colorectal, endometrial and other types of cancer [8–12]. Early age of onset and familial clustering of cancers are clinical indicators of LS. In order to identify families suspected of having LS, the Amsterdam criteria and Bethesda guidelines were established [13–16]. An essential characteristic of LS is MMR deficiency (dMMR) of the tumours, as shown by reduced or lost immunohistochemical staining and/or microsatellite instability (MSI). A previous study by our group showed that in families meeting the Amsterdam or Bethesda criteria and having microsatellite instability-high colorectal cancer, only 53% carried a pathogenic germline MMR or *EPCAM* gene variant [17]. It has been suggested to designate individuals from families without a pathogenic germline MMR gene defect despite signs of dMMR as having Lynch-like syndrome (LLS) [18, 19]. In our previous study, it could be shown that in 37% of Amsterdam-II positive families no signs of dMMR could be found [17]. Individuals from Amsterdam-positive families without any signs of dMMR were characterised as a distinct risk group, the so-called “familial colorectal cancer type X” (FCCTX or FCCX) [18, 20]. The different clinical aspects and molecular features of LS, LLS, and FCCX are summarized elsewhere [21].

A comprehensive characterisation of cancer risks is an important prerequisite for developing appropriate cancer surveillance and prevention programmes. To date, many studies have characterised cancer risks in LS, also using prospective data, but fewer studies have characterised cancer risks in LLS and FCCX [7–12, 19–33]. The aim of the present analysis was therefore to estimate and compare the risk for different types of cancers in individuals with LS, LLS, and FCCX based on data from a prospective surveillance study of the German Consortium for Familial Intestinal Cancer. Additionally, we aimed to compare these risk estimations to cancer risks in the general population in Germany.

## Methods

### Study population

The study population was taken from a prospective registry study of the “German Consortium for Familial Intestinal Cancer” (formerly termed “German HNPCC Consortium”). Six university centres collected information about families suspected of having Lynch syndrome based on the Amsterdam-II criteria and/or revised Bethesda guidelines [15, 16]. All participants gave their written informed consent at registry inclusion, and the registry was approved by the Ethics Committees of all participating institutions.

A tissue sample (tumour or adenoma) of the index patient was examined for MMR deficiency (dMMR) using immunohistochemistry (IHC) and/or microsatellite analysis (MSA). In case of dMMR (or if no tissue sample was available), a germline mutation analysis of the MMR genes *MLH1*, *MSH2*, *MSH6* and *PMS2,* and the *EPCAM* gene was carried out. Details about the diagnostic procedure are described elsewhere [17]. According to the results of the tissue and subsequent germline DNA analyses, index patients were classified as having LS (i.e. with a proven class 4/5 germline variant), LLS (i.e. fulfilling the Amsterdam-II and/or revised Bethesda criteria and not having a class 4/5 germline variant despite signs of dMMR in at least one family member), and FCCX (i.e. not having any signs of dMMR in the family while fulfilling the Amsterdam-II criteria). Relatives of LS index patients, who were predictively tested for the specific class 4/5 variant found in the index patient, were also considered as having LS if they were tested positive. Relatives of LLS and FCCX index patients were also considered as having LLS and FCCX, respectively. Individuals from families with dMMR due to *MLH1* methylation were not regarded as having LLS.

Individuals with LS, LLS, or FCCX according to the above definitions were invited to participate in an intensified surveillance programme comprising annual colonoscopies, esophagogastroduodenoscopies, and gynaecological examinations. These individuals comprised both index patients and at-risk relatives. They were prospectively followed up and the result of each single surveillance examination was recorded in the registry.

Individuals were included in the present analysis if they had LS, LLS, or FCCX according to the above definitions. In the LS group, only *MLH1*, *MSH2*, and *MSH6* carriers were included, whereas *EPCAM* and *PMS2* carriers were excluded due to the low sample size. Additionally, all individuals had to have at least one colonoscopy after study registration and a prospective observation time of more than half a year.

### Statistical analysis

From the above study population, eight different cohorts were defined and cancer risks were determined for the following eight types of cancers according to the International Classification of Diseases (ICD-10): any cancer (all Cxx without C77-C79), colorectal (C18-C20), stomach (C16), small bowel (C17), urothelial (C65-C68), female breast (C50), ovarian (C56), and endometrial cancer (C54.1). For each cancer type, its first incident occurrence was analysed. Individuals, who already had the cancer of interest before or within half a year after the start of prospective observation (prevalent cancers) were excluded. Therefore, patient numbers and observation times were different between the eight cohorts. Patients with prevalent cancers other than the cancer type of interest were not excluded. Females who had undergone a hysterectomy prior to the start of the prospective observation were excluded from the endometrial cancer and females with an oophorectomy from the ovarian cancer analyses.

Prospective observation started at the first colonoscopy after study registration or at age 25, whichever occurred last. Observation ended at the specific cancer event of interest, the age of 80, the last documented contact before May 12, 2019, or death, whichever came first. For calculations of endometrial cancer, hysterectomy was an additional reason for the end of observation, as well as oophorectomy for ovarian cancer. The incident occurrence of cancers other than the cancer type of interest was no reason for censoring [9, 10]. Cumulative cancer risks were determined for the different cancer types stratified by the three risk groups LS, LLS and FCCX, and by subgrouping the LS group into *MLH1*, *MSH2* and *MSH6* carriers. Risk estimation was done using the Kaplan-Meier product limit estimator accounting for the age at the beginning of prospective observation (left-truncation) [34]. For comparisons between groups, the log-rank test was used. The proportional hazards assumption was tested using scaled Schoenfeld residuals [35, 36]. In addition, the log-minus-log-transformed 95% confidence intervals of the product limit estimator were determined [34, 37]. In order to compare cancer risks with the general population, the standardised incidence ratio (SIR) was calculated, which is defined as the ratio between the observed number of cancers of interest and the expected number of cancers of interest in the general population. The number of expected cancers of interest was determined by the sum of all products of the age-specific incidence rates for the general population with the corresponding person-years. The source of age-specific incidence rates in 5-year-intervals for the general population in Germany (2000–2014) was the German Centre for Cancer Registry Data of the Robert Koch-Institute (Berlin, Germany. URL: www.krebsdaten.de/abfrage). As information regarding skin malignancies except malignant melanoma was not available for the general population, individuals with such neoplasms were excluded from the SIR calculation of the cancer type “any cancer”. Ninety-five percent confidence intervals for SIRs were calculated assuming a Poisson distribution.

All reported testing was two-sided, and *p*-values lower than 0.05 were considered statistically significant. Statistical analyses were carried out with R 3.4.2 for Windows (R Core Team. R: A language and environment for statistical computing. R Foundation for Statistical Computing, Vienna, Austria. URL: https://www.R-project.org).

## Results

### Patient characteristics

The study population comprised a total of 1830 patients, including 594 in the LLS, 116 in the FCCX group, and 1120 in the LS group (447 *MLH1*, 549 *MSH2*, and 124 *MSH6* carriers). In total, 1200 individuals were index patients. The detailed patient characteristics for the eight different cancer types are summarised in Table 1 (a sex specific summary is shown in Additional file 1: Tables S1 and S2). The number of individuals at risk for first incident cancer ranged from 322 to 1102 in LS, 120 to 586 in LLS, and 40 to 116 in FCCX, depending on the cancer type. The median age at the start of the prospective observation ranged from 35 to 49 years depending on the risk group and cancer of interest. Table S3 in Additional file 1 shows a detailed summary of patient numbers, observation times and numbers of incident cancers. The types of cancers considered for the calculation of the type “any cancer” are given in Additional file 1: Table S4.

**Table 1:** Patient characteristics

	**FCCX**	**LLS**^**a**^	**LS**			**Total**
			***MLH1***	***MSH2***	***MSH6***	
	*n* = 116	*n* = 594	*n* = 447	*n* = 549	*n* = 124	*n* = 1830
[Index patients]	[79]	[451]	[279]	[324]	[67]	[1200]
**Individuals at risk, number [index patients]**						
Any	40 [9]	120 [11]	116 [6]	158 [12]	48 [5]	482 [43]
Colorectal	42 [10]	140 [25]	133 [14]	206 [36]	57 [6]	578 [91]
Stomach	115 [78]	584 [442]	439 [271]	539 [316]	124 [67]	1801 [1174]
Small bowel	116 [79]	586 [443]	429 [263]	536 [315]	124 [67]	1791 [1167]
Urothelial	116 [79]	585 [443]	445 [277]	524 [304]	122 [67]	1792 [1170]
Female breast	58 [40]	307 [224]	214 [115]	256 [138]	60 [28]	895 [545]
Ovarian	56 [38]	270 [190]	187 [98]	202 [105]	48 [22]	763 [453]
Endometrial	53 [36]	254 [186]	152 [76]	163 [83]	45 [19]	667 [400]
**Age at start of prospective observation, median (interquartile range)**						
Any	40 (35–50)	39 (30–46)	35 (29–43)	36 (30–43)	40 (34–48)	38 (30–45)
Colorectal	40 (35–51)	41 (31–48)	37 (30–45)	39 (31–48)	42 (36–51)	39 (31–48)
Stomach	49 (41–55)	44 (38–51)	44 (35–54)	44 (36–52)	44 (37–57)	45 (37–53)
Small bowel	49 (41–55)	44 (38–52)	44 (35–53)	44 (36–52)	44 (37–57)	45 (37–53)
Urothelial	49 (41–55)	44 (38–51)	44 (35–54)	44 (36–52)	44 (37–56)	44 (37–53)
Female breast	48 (41–55)	44 (38–52)	44 (35–54)	44 (36–54)	44 (38–55)	44 (37–53)
Ovarian	48 (41–53)	44 (38–50)	41 (34–52)	42 (34–51)	40 (37–49)	43 (36–51)
Endometrial	49 (41–55)	43 (37–49)	38 (32–47)	40 (33–46)	40 (37–47)	41 (35–49)
**Median follow-up time, person-years**						
Any	6.8	6.9	6.5	6.4	6.5	6.5
Colorectal	7.1	6.3	6.5	7.0	7.1	6.8
Stomach	7.1	6.5	7.3	7.8	6.7	7.1
Small bowel	7.1	6.4	7.2	7.7	6.7	7.0
Urothelial	7.1	6.4	7.2	7.5	6.7	6.9
Female breast	7.2	6.5	6.7	8.1	7.1	7.1
Ovarian	7.3	6.3	6.5	7.0	7.3	6.8
Endometrial	7.2	5.9	6.0	6.0	7.1	6.1
**Cumulative follow-up time, person-years**						
Any	308	838	819	1105	326	3395
Colorectal	329	964	938	1509	417	4157
Stomach	854	4019	3300	4222	843	13239
Small bowel	858	4011	3212	4164	843	13088
Urothelial	858	4003	3314	4006	829	13010
Female breast	420	2093	1539	1963	451	6467
Ovarian	417	1805	1292	1397	368	5278
Endometrial	376	1581	994	1019	315	4285
**Individuals with incident cancer, number**						
Any	2	8	13	27	3	53
Colorectal	0	6	7	19	2	34
Stomach	0	4	4	5	0	13
Small bowel	0	1	9	14	0	24
Urothelial	1	4	9	19	0	33
Female breast	1	3	3	6	4	17
Ovarian	0	0	0	5	0	5
Endometrial	0	5	9	13	1	28

^**a**^*n* = 320 with deficient MMR protein expression in MLH1, *n* = 127 in MSH2, *n* = 26 in MSH6. IHC was not performed in *n* = 121 individuals

### Comparison of cancer risks between LS, LLS, and FCCX

**Fig. 1:**
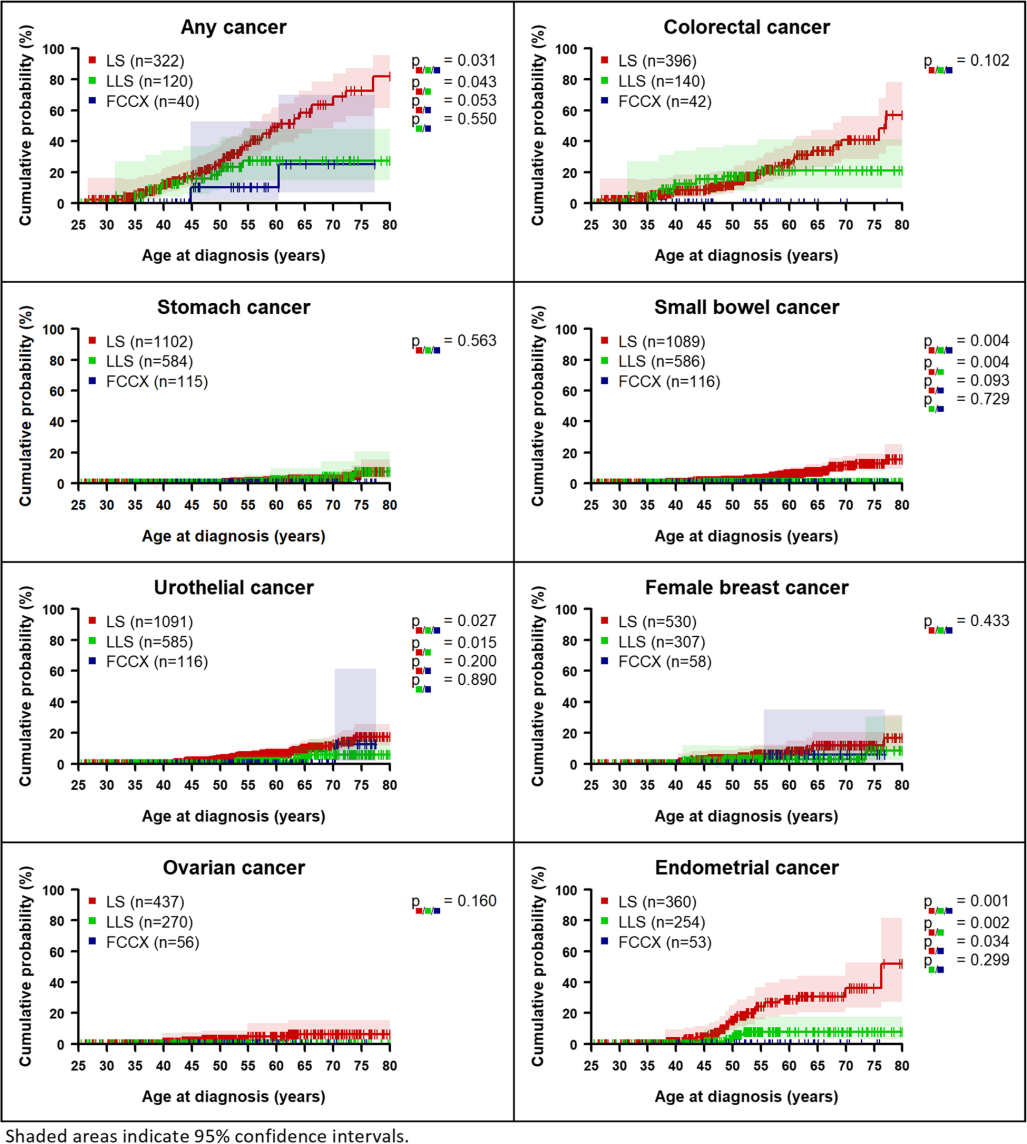
Age-dependent cumulative cancer risks

The risk for any cancer by the age of 70 years was 63.7% (95%CI 48.5–78.8%) for LS, 27.3% (95%CI 14.5–47.8%) for LLS, and 25.0% (95%CI 6.6–70.2%) for FCCX patients (Fig. 1 and Table 2). Cumulative cancer risk was higher for the LS group than for LLS or FCCX for any cancer, small bowel, urothelial, female breast, ovarian, and endometrial cancer. The risks in the FCCX group were lower than in the LLS group for all cancer types, except for female breast and ovarian cancer. The risk of any cancer, small bowel, urothelial, and endometrial cancer was statistically significantly higher in the LS group than in the LLS group. No statistically significant differences for “any cancer” were found in the sex-specific analyses, whereas significant differences were also observed in males for small bowel and urothelial cancer, but not in females. No incident colorectal cancer events were observed in the FCCX group. LLS patients showed a lower colorectal cancer risk (21.0%, 95%CI 9.9–41.3%) than LS patients (40.9%, 95%CI 28.3–56.4%) at age 70, but the difference between LS, LLS, and FCCX was not statistically significant (*p* = 0.102). There were no significant differences between the LLS and FCCX groups (Additional file 1: Figure S7).

**Table 2:** Cumulative cancer risks (%) by age

					**LS**		
**Cancer type**	**Age (years)**	**FCCX**	**LLS**	**LS**	***MLH1***	***MSH2***	***MSH6***
Any	40	0.0 (−)	12.5 (4.2–34.1)	12.2 (6.4–22.5)	14.0 (5.5–33.1)	13.8 (5.7–31.4)	0.0 (−)
	50	10.0 (1.5–52.7)	20.5 (9.7–40.4)	25.6 (17.5–36.4)	24.1 (12.8–42.6)	31.6 (19.5–48.5)	7.7 (1.1–43.4)
	60	10.0 (1.5–52.7)	27.3 (14.5–47.8)	49.0 (37.5–61.9)	44.0 (26.6–66.3)	60.3 (44.0–77.1)	20.9 (5.3–63.3)
	70	25.0 (6.6–70.2)	27.3 (14.5–47.8)	63.7 (48.5–78.8)	52.0 (31.9–75.3)	72.2 (53.2–88.4)	36.7 (12.7–78.6)
Colorectal	40	0.0 (−)	12.4 (4.2–33.8)	8.4 (3.7–18.3)	10.0 (3.3–27.9)	9.5 (2.9–28.3)	0.0 (−)
	50	0.0 (−)	17.4 (7.5–37.4)	11.8 (6.3–21.5)	10.0 (3.3–27.9)	16.3 (7.7–32.7)	0.0 (−)
	60	0.0 (−)	21.0 (9.9–41.3)	25.4 (16.8–37.2)	23.4 (10.8–46.6)	33.7 (21.0–51.1)	0.0 (−)
	70	0.0 (−)	21.0 (9.9–41.3)	40.9 (28.3–56.4)	30.4 (14.8–55.8)	49.1 (32.9–68.1)	14.3 (2.1–66.6)
Stomach	40	0.0 (−)	0.0 (−)	0.0 (−)	0.0 (−)	0.0 (−)	0.0 (−)
	50	0.0 (−)	0.0 (−)	0.0 (−)	0.0 (−)	0.0 (−)	0.0 (−)
	60	0.0 (−)	2.4 (0.6–9.9)	1.7 (0.6–4.6)	0.9 (0.1–6.4)	2.4 (0.8–7.4)	0.0 (−)
	70	0.0 (−)	4.6 (1.4–14.5)	2.9 (1.3–6.5)	2.5 (0.6–9.9)	3.6 (1.3–9.5)	0.0 (−)
Small bowel	40	0.0 (−)	0.0 (−)	0.6 (0.1–4.1)	1.4 (0.2–9.8)	0.0 (−)	0.0 (−)
	50	0.0 (−)	0.6 (0.1–4.4)	1.8 (0.7–4.8)	1.4 (0.2–9.8)	2.5 (0.8–7.7)	0.0 (−)
	60	0.0 (−)	0.6 (0.1–4.4)	5.9 (3.5–10.1)	5.0 (1.9–12.8)	7.6 (4.0–14.2)	0.0 (−)
	70	0.0 (−)	0.6 (0.1–4.4)	11.7 (7.6–17.6)	11.5 (5.8–22.0)	13.9 (8.0–23.4)	0.0 (−)
Urothelial	40	0.0 (−)	0.0 (−)	0.0 (−)	0.0 (−)	0.0 (−)	0.0 (−)
	50	0.0 (−)	0.0 (−)	2.7 (1.3–5.6)	0.9 (0.1–6.1)	4.7 (2.1–10.3)	0.0 (−)
	60	0.0 (−)	1.5 (0.4–5.9)	6.5 (4.0–10.4)	5.7 (2.4–13.3)	8.4 (4.7–14.7)	0.0 (−)
	70	0.0 (−)	5.7 (2.0–16.0)	12.5 (8.3–18.5)	11.3 (5.6–22.0)	16.3 (9.9–26.2)	0.0 (−)
Female breast	40	0.0 (−)	0.0 (−)	0.0 (−)	0.0 (−)	0.0 (−)	0.0 (−)
	50	0.0 (−)	2.9 (0.7–11.4)	3.4 (1.3–8.7)	2.0 (0.3–13.6)	1.6 (0.2–10.6)	12.0 (3.1–40.6)
	60	5.9 (0.9–35.0)	2.9 (0.7–11.4)	7.9 (4.2–14.7)	7.3 (2.4–21.2)	6.8 (2.6–17.1)	12.0 (3.1–40.6)
	70	5.9 (0.9–35.0)	2.9 (0.7–11.4)	11.9 (6.8–20.3)	7.3 (2.4–21.2)	12.2 (5.5–25.8)	20.8 (7.0–53.0)
Ovarian	40	0.0 (−)	0.0 (−)	1.0 (0.1–7.1)	0.0 (−)	2.4 (0.3–16.1)	0.0 (−)
	50	0.0 (−)	0.0 (−)	2.9 (0.9–8.7)	0.0 (−)	6.8 (2.2–19.6)	0.0 (−)
	60	0.0 (−)	0.0 (−)	4.9 (1.7–13.3)	0.0 (−)	10.7 (4.0–26.9)	0.0 (−)
	70	0.0 (−)	0.0 (−)	6.4 (2.6–15.5)	0.0 (−)	14.0 (5.9–31.2)	0.0 (−)
Endometrial	40	0.0 (−)	0.0 (−)	1.4 (0.2–9.3)	0.0 (−)	3.1 (0.4–20.2)	0.0 (−)
	50	0.0 (−)	4.3 (1.4–12.8)	15.3 (9.1–25.0)	14.5 (6.8–29.6)	16.8 (7.9–33.8)	10.0 (1.5–52.7)
	60	0.0 (−)	7.7 (3.2–17.6)	28.8 (19.1–41.9)	19.2 (9.2–37.5)	40.1 (24.5–60.7)	10.0 (1.5–52.7)
	70	0.0 (−)	7.7 (3.2–17.6)	36.1 (23.4–52.7)	35.4 (13.8–72.2)	44.1 (27.8–64.5)	10.0 (1.5–52.7)

Numbers in brackets are 95% confidence intervals

**Fig. 2:**
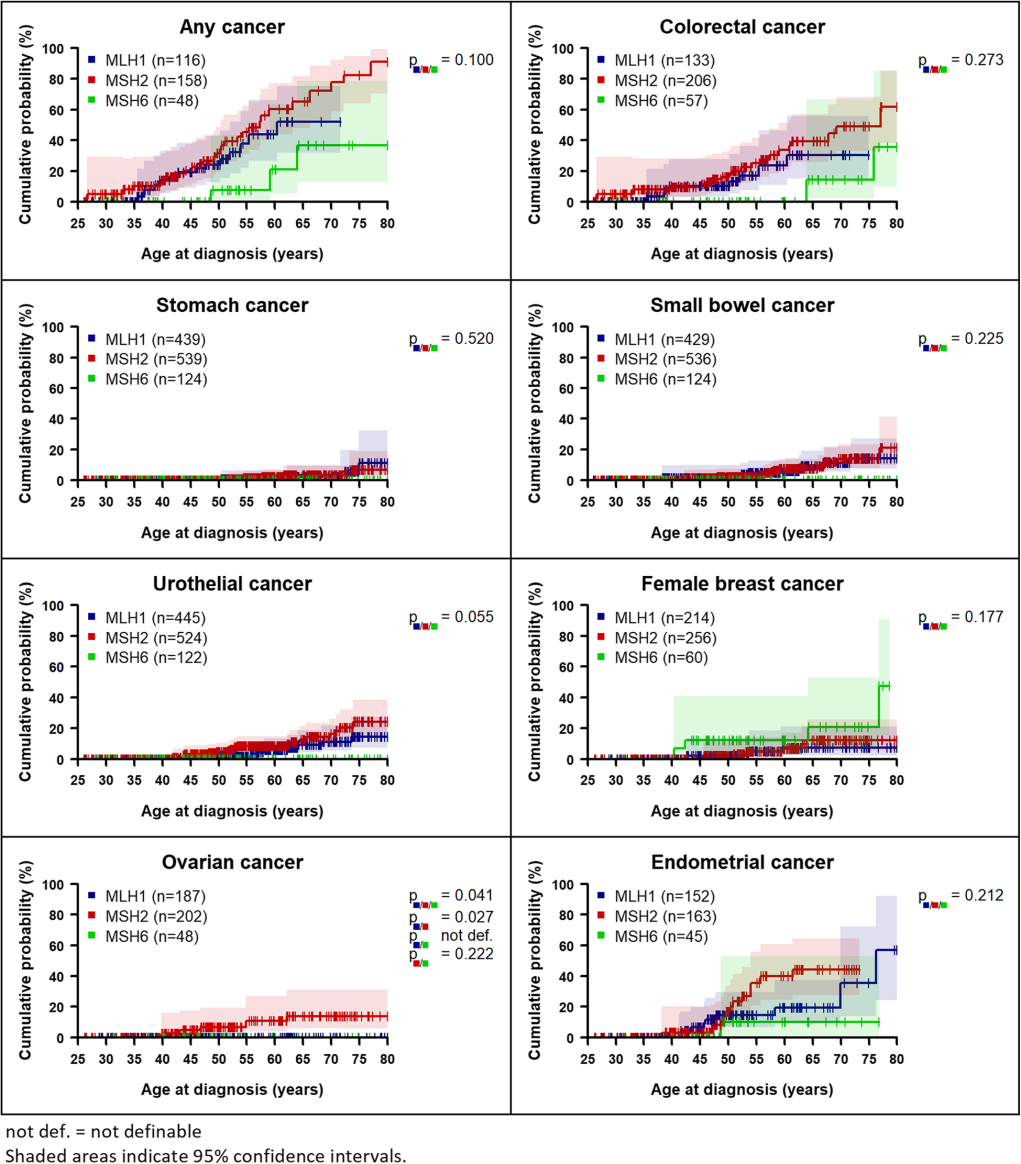
Age-dependent cumulative cancer risks of LS patients by gene

Within the LS group, higher cancer risks were found for *MLH1* and *MSH2* carriers than for *MSH6* carriers for any cancer, colorectal, stomach, small bowel, urothelial, and endometrial cancer (Fig. 2 and Table 2, Additional file 1: Figure S8). The risk of ovarian cancer was highest in *MSH2* carriers (Additional file 1: Tables S5 and S6) with statistically significant differences between the three genes (*p* = 0.041). For urothelial cancer, risk differences were borderline non-significant (*p* = 0.055).

The LLS group comprised 320 individuals from families with MMR deficiency in the MLH1 protein, 127 in MSH2, and 26 in MSH6. The remaining 121 patients could not be assigned to a specific MMR protein, because only MSA but not IHC had been performed. There were no statistically significant differences in cancer risks between MLH1, MSH2 and MSH6 in the LLS group except for urothelial cancer in women (*p* = 0.003). Women from LLS families with MSH2 MMR deficiency had a higher risk for urothelial cancer compared to women from families with MMR deficiency in MLH1 and MSH6.

Moreover, we found significant differences in cancer risks between index patients and the at-risk relatives for colorectal and endometrial cancer in the LLS group. Index patients had a higher risk (*p* = 0.027) of colorectal cancer at age 70 (50.0%, 95%CI 19.6–88.9%) compared to their relatives (13.2%, 95%CI 4.4–35.9%), but a lower risk (*p* = 0.010) of endometrial cancer (4.0%, 95%CI 1.0–15.3% vs. 23.1%, 95%CI 8.1–55.8%).

### Comparison with general population risks

**Fig. 3:**
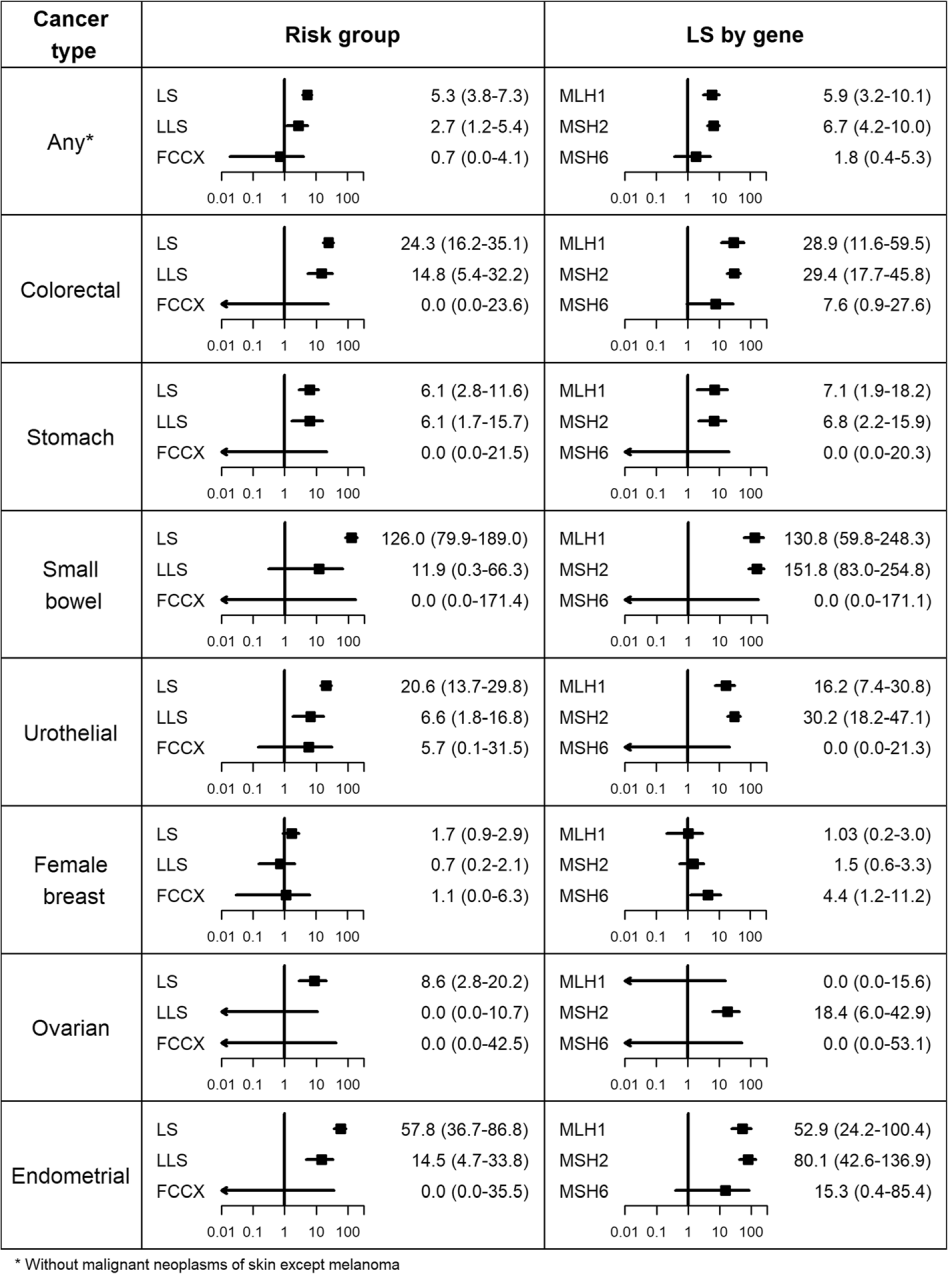
Comparison with general population risks: standardised incidence ratios (SIRs) with 95% confidence intervals

Compared to the general population, cancer risks in the LS group were higher for all cancer types. These results were statistically significant for all cancer types regardless of sex, with the exception of female breast cancer (Fig. 3, Additional file 1: Figures S9 and S10). Standardised incidence ratios (SIRs) ranged from 5.3 for “any cancer” to 126.0 for small bowel cancer. Increased SIRs in *MLH1* and *MSH2* carriers were found in male LS patients for all types of cancer. These results were also statistically significant, except for stomach cancer where only *MLH1* carriers had a significantly increased risk. The SIR of colorectal cancer was also elevated in male *MSH6* carriers, although not showing statistical significance. Female *MLH1* and *MSH2* carriers had elevated SIRs for any cancer, colorectal, stomach, small bowel, urothelial, and endometrial cancer, which were all statistically significant, with the exception of stomach cancer in *MLH1* carriers. In addition, female *MSH2* carriers showed a significantly increased risk of ovarian cancer. The SIRs for female breast cancer were only slightly marginally elevated for *MLH1* and *MSH2* carriers compared to the general population, while the SIR for *MSH6* carriers was statistically significantly higher.

Larger SIRs were observed in the LLS group for any cancer, colorectal, stomach, small bowel, urothelial, and endometrial cancer, which were statistically significant except for small bowel cancer. Regarding sex, significantly increased SIRs in the LLS group were detected in females for endometrial and urothelial cancer, as well as in males for colorectal and stomach cancer. Cancer risks were not statistically significantly increased in the FCCX group compared to the general population.

## Discussion

In this prospective study, we investigated different organ-specific cancer risks in Lynch syndrome, Lynch-like syndrome, and familial colorectal cancer type X. To the best of our knowledge, this is the first report comparing cumulative cancer incidences in these three risk groups based on prospective follow-up data. We found higher cancer risks for LS than for LLS and FCCX patients. Some cancer risks, such as colorectal and endometrial cancer, tended to be higher in LLS than in the FCCX group, but this did not reach statistical significance.

A large number of retro- and prospective studies have investigated cancer risks in LS patients [7–12, 19, 22–33]. The largest prospective studies to date, based on internationally pooled data gathered in the Prospective Lynch Syndrome Database (PLSD), provided risk estimates for various cancer types in LS patients stratified by age, gene, and sex [9–12]. The cumulative risks in LS patients found in our study are in agreement with those PLSD studies, which did not include the data of our present study [9–11]. We found statistically significant risk differences between *MLH1*, *MSH2,* and *MSH6* carriers only for ovarian cancer, where *MSH2* carriers had the highest risk. This agrees with the results from the PLSD and other studies showing that *MSH2* carriers have higher ovarian cancer risks than *MLH1* or *MSH6* carriers [25, 26]. Compared to the general population, we found a statistically significantly increased risk of female breast cancer (SIR = 4.4, 95%CI 1.2–11.2) only in *MSH6* carriers. This is in agreement with results from a retrospective study of Roberts et al. [38], but in disagreement with others studies, which have reported either no increased risks for LS patients or elevated risks in *MLH1* carriers [28, 29].

Compared to LS, considerably fewer studies have investigated cancer risks in LLS and FCCX. Rodriguez-Soler and colleagues performed a population-based study with 1705 consecutively included colorectal cancer patients comparing cancer risks between LS, LLS, and sporadic cancer [19]. They found that the risk of cancer in LLS is lower compared to LS, but higher compared to sporadic cancer. In our study, we found significantly lower cancer risks in LLS compared to LS for any cancer, small bowel, urothelial, and endometrial cancer. However, compared to the general population, the risks of any cancer, colorectal, stomach, urothelial, and endometrial cancer were significantly elevated for LLS as well as for LS. This is of particular interest, as LLS patients may need specific surveillance programmes. It has been suggested that individuals with a history of cancer, as well as their first-degree relatives should undergo colonoscopic screening every 3 years [39]. Regular esophagogastroduodenoscopy might also add value, as our study showed an increased risk of stomach and small bowel cancer. However, it should be noted that the LLS group is heterogeneous as it may contains both individuals with sporadic tumours with somatic MMR variants and individuals with MMR germline variants, which were not detected.

We did not find any significant differences in cancer risks between MLH1, MSH2 and MSH6 in the LLS group with the exception of urothelial cancer in women, where women from families with a MMR deficiency in the MSH2 protein showed a higher risk. There were also no significant differences of cancer risks between index patients and the at-risk relatives in the LLS group, except for colon cancer and endometrial cancer, where index patients had a higher risk of colon cancer but a lower risk of endometrial cancer compared to the at-risk relatives. These results need to be validated in larger, ideally international collaborative studies, before specific screening recommendations can be made for this risk group.

Lindor et al. compared the incidence of different cancer types in individuals from Amsterdam-‍I families with and without dMMR [20]. They concluded that individuals without dMMR in their family have a lower incidence of cancer than those with dMMR. Choi et al. compared the risks of first and metachronous colorectal cancers in LS with those of members of FCCX families and found higher risks in individuals with LS compared to FCCX family members [23]. We observed lower cancer risks for FCCX compared to LS and LLS groups, although these were not statistically significant. In addition, we found no significantly higher risks for individuals of the FCCX group compared with the general population. This raises the question of whether FCCX patients require a different surveillance programme compared to LS and LLS patients, and whether screening similar to the general population would be sufficient for this group. Analyses with a larger number of FCCX individuals would be helpful in order to investigate this further.

A major strength of the present study was its prospective design, which mitigates the problem of overestimation of cancer risks due to ascertainment bias in clinic-based retrospective studies [40]. However, some limitations also need to be noted. One was the comparably low sample size and observation time in the FCCX group, resulting in large confidence intervals of the risk estimates. Secondly, observation times above the age of 60 years were comparably low. Thirdly, since all of these patients were under intensified colonoscopic surveillance, with possible colorectal cancer prevention (to an unknown extent) due to adenoma removal, the colorectal cancer risk estimates obtained in our study do not reflect the natural course of disease. Fourthly, there could be some underestimation of SIRs for endometrial cancer since general population incidences were only available for the ICD-10 group C54 and not specifically for C54.1.

## Conclusions

This prospective study provides data on cancer risk estimation in patients with LS and particularly with LLS and FCCX, which is important to develop appropriate surveillance programmes for these specific intermediate risk groups. However, before specific surveillance recommendations can be given, larger prospective studies are needed to obtain more precise risk estimates. We propose international databases for LLS and FCCX, similar to the Prospective Lynch Syndrome Database (PLSD), be set up.

## Supplementary information

**Additional file 1 Table S1.** Characteristics of female patients. **Table S2.** Characteristics of male patients. **Table S3.** Number of patients, observation times (person-years) and number of incident cancers. **Table S4.** Types of incident cancers considered as “any cancer”. **Table S5.** Cumulative cancer risks (%) by age for female patients. **Table S6.** Cumulative cancer risks (%) by age for male patients. **Figure S7.** Age-dependent cumulative cancer risks by risk group and sex. **Figure S8.** Age-dependent cumulative cancer risks of LS patients by gene and sex. **Figure S9.** Comparison with general population risks: standardised incidence ratios (SIRs) with 95% confidence interval for female patients. **Figure S10.** Comparison with general population risks: standardised incidence ratios (SIRs) with 95% confidence interval for male patients.

## Data Availability

The datasets used and analysed during the current study are available from the corresponding author on reasonable request.

## Abbreviations

CI: Confidence interval
dMMR: Mismatch repair deficiency
FCCX: Familial colorectal cancer type X
ICD: International Statistical Classification of Diseases and Related Health Problems
IHC: Immunohistochemistry
LLS: Lynch-like syndrome
LS: Lynch syndrome
MMR: Mismatch repair
MSA: Microsatellite analysis
MSI: Microsatellite instability
SIR: Standardised incidence ratio
