# Supplementary material for: Cancer risks in Lynch syndrome, Lynch-like syndrome, and familial colorectal cancer type X: a prospective cohort study
Source: BMC Cancer. 2020 May 24;20:460. doi: 10.1186/s12885-020-06926-x (PMC7245918; doi:10.1186/s12885-020-06926-x)
Supplement: Supplementary file 1 — Additional file 1 Table S1. Characteristics of female patients. Table S2. Characteristics of male patients. Table S3. Number of patients, observation times (person-years) and number of incident cancers. Table S4. Types of incident cancers considered as “any cancer”. Table S5. Cumulative cancer risks (%) by age for female patients. Table S6. Cumulative cancer risks (%) by age for male patients. Figure S7. Age-dependent cumulative cancer risks by risk group and sex. Figure S8. Age-dependent cumulative cancer risks of LS patients by gene and sex. Figure S9. Comparison with general population risks: standardised incidence ratios (SIRs) with 95% confidence interval for female patients. Figure S10. Comparison with general population risks: standardised incidence ratios (SIRs) with 95% confidence interval for male patients. [file 12885_2020_6926_MOESM1_ESM.pdf]

Additional file 1

Title: Cancer risks in Lynch syndrome, Lynch-like syndrome, and familial colorectal cancer type X: A prospective cohort study

Karolin Bucksch, Silke Zachariae, Stefan Aretz, Reinhard Büttner, Elke Holinski-Feder, Stefanie Holzapfel, Robert Hüneburg, Matthias Kloor, Magnus von Knebel Doeberitz, Monika Morak, Gabriela Möslein, Jacob Nattermann, Claudia Perne, Nils Rahner, Wolff Schmiegel, Karsten Schulmann, Verena Steinke-Lange, Christian P. Strassburg, Deepak B. Vangala, Jürgen Weitz, Markus Loeffler, Christoph Engel

on behalf of the German Consortium for Familial Intestinal Cancer

Supplemental information

Table S1:	Characteristics of female patients.	page 2
Table S2:	Characteristics of male patients.	page 3
Table S3:	Number of patients, observation times (person-years) and number of incident cancers.	page 4
Table S4:	Types of incident cancers considered as "any cancer".	page 5
Table S5:	Cumulative cancer risks (%) by age for female patients.	page 6
Table S6:	Cumulative cancer risks (%) by age for male patients.	page 7
Figure S7:	Age-dependent cumulative cancer risks by risk group and sex.	page 8
Figure S8:	Age-dependent cumulative cancer risks of LS patients by gene and sex.	page 9
Figure S9:	Comparison with general population risks: standardised incidence ratios (SIRs) with 95% confidence interval for female patients.	page 10
Figure S10:	Comparison with general population risks: standardised incidence ratios (SIRs) with 95% confidence interval for male patients.	page 11

Table S1: Characteristics of female patients.

	**FCCX**	**LLS**	**LS**			**Total**
			MLH1	***MSH2***	***MSH6***	
	n=59	n=315	n=222	n=265	n=63	n=924
**Individuals at risk, number**						
Any	21	77	72	86	28	284
Colorectal	22	94	86	123	35	360
Stomach	59	312	220	261	63	915
Small bowel	59	310	218	260	63	910
Urothelial	59	312	221	255	62	909
**Age at start of prospective observation, median (interquartile range)**						
Any	41 (36-55)	39 (29-48)	37 (28-41)	36 (30-43)	40 (38-46)	38 (31-45)
Colorectal	41 (36-54)	42 (32-52)	38 (29-46)	41 (33-50)	42 (39-50)	40 (33-50)
Stomach	48 (41-55)	44 (38-52)	44 (35-55)	45 (36-54)	45 (38-57)	45 (37-54)
Small bowel	48 (41-55)	44 (38-52)	44 (35-55)	45 (36-54)	45 (38-57)	45 (37-54)
Urothelial	48 (41-55)	44 (38-52)	44 (35-55)	44 (36-53)	44 (38-57)	45 (37-54)
**Median follow-up time, person-years**						
Any	8.3	7.1	6.6	6.6	6.8	6.9
Colorectal	8.6	7.0	6.6	7.3	7.1	7.0
Stomach	7.3	6.5	6.7	8.1	7.4	7.1
Small bowel	7.3	6.5	6.5	8.0	7.4	7.1
Urothelial	7.3	6.4	6.6	7.9	7.5	7.0
**Cumulative follow-up time, person-years**						
Any	175	539	521	576	209	2020
Colorectal	187	652	617	884	286	2626
Stomach	439	2144	1573	2016	477	6649
Small bowel	439	2119	1542	2006	477	6583
Urothelial	439	2120	1566	1935	474	6534
**Individuals with incident cancer, number**						
Any	2	5	8	15	3	33
Colorectal	0	2	2	12	1	17
Stomach	0	1	1	3	0	5
Small bowel	0	1	4	5	0	10
Urothelial	0	3	2	7	0	12

Table S2: Characteristics of male patients.

	FCCX	LLS	LS			Total
			MLH1	*MSH2*	*MSH6*	
	n=57	n=279	n=225	n=284	n=61	n=906
**Individuals at risk, number**						
Any	19	43	44	72	20	198
Colorectal	20	46	47	83	22	218
Stomach	56	272	219	278	61	886
Small bowel	57	276	211	276	61	881
Urothelial	57	273	224	269	60	883
**Age at start of prospective observation, median (interquartile range)**						
Any	39 (34-47)	39 (30-43)	34 (30-44)	36 (29-43)	37 (31-58)	36 (30-44)
Colorectal	39 (34-48)	40 (30-45)	35 (30-44)	37 (30-46)	40 (31-59)	37 (31-46)
Stomach	49 (41-56)	44 (38-51)	44 (35-53)	44 (36-51)	44 (34-55)	44 (37-52)
Small bowel	49 (41-56)	44 (38-51)	44 (35-52)	44 (36-52)	44 (34-55)	44 (37-52)
Urothelial	49 (41-56)	44 (38-51)	44 (36-53)	44 (36-51)	44 (34-54)	44 (37-52)
**Median follow-up time, person-years**						
Any	6.6	6.3	5.7	6.3	3.8	6.2
Colorectal	6.8	5.8	6.0	6.6	5.4	6.3
Stomach	7.0	6.3	7.8	7.5	4.2	7.0
Small bowel	7.0	6.3	7.8	7.2	4.2	6.9
Urothelial	7.0	6.3	7.7	7.1	4.1	6.9
**Cumulative follow-up time, person-years**						
Any	133	299	298	529	116	1375
Colorectal	142	312	320	625	132	1531
Stomach	415	1875	1728	2207	366	6591
Small bowel	419	1893	1670	2158	366	6506
Urothelial	419	1883	1749	2071	355	6476
**Individuals with incident cancer, number**						
Any	0	3	5	12	0	20
Colorectal	0	4	5	7	1	17
Stomach	0	3	3	2	0	8
Small bowel	0	0	5	9	0	14
Urothelial	1	1	7	12	0	21

Table S3: Number of patients, observation times (person-years) and number of incident cancers.

Cancer type	Age group	FCCX			LLS			LS								
								MLH1			*MSH2*			*MSH6*		
		No*	PY[$]	EV[§]	No*	PY[$]	EV[§]	No*	PY[$]	EV[§]	No*	PY[$]	EV[§]	No*	PY[$]	EV[§]
Any[#]	25-<30	3	10	0	30	64	0	31	99	0	38	111	1	4	7	0
	30-<40	18	74	0	56	235	3	67	262	4	87	364	3	22	68	0
	40-<50	23	97	0	59	297	3	55	315	4	80	344	7	23	127	1
	50-<60	18	80	0	42	158	2	34	121	4	40	166	8	17	78	1
	60-<70	8	33	1	12	56	0	9	21	1	15	57	2	10	30	1
	70-<80	2	8	0	9	27	0	1	2	0	5	24	2	3	15	0
	Total	39	301	1	120	838	8	116	819	13	154	1066	23	48	326	3
Colorectal	25-<30	3	10	0	30	64	0	33	106	0	39	112	1	4	7	0
	30-<40	20	76	0	58	237	3	70	270	3	96	387	2	22	68	0
	40-<50	25	107	0	67	327	2	61	349	0	108	495	4	26	154	0
	50-<60	20	89	0	51	198	1	46	163	3	76	300	7	24	104	0
	60-<70	9	40	0	17	79	0	15	45	1	35	162	4	15	49	1
	70-<80	2	8	0	15	59	0	5	6	0	13	53	1	7	35	1
	Total	42	329	0	140	964	6	133	938	7	206	1509	19	57	417	2
Stomach	25-<30	4	11	0	46	101	0	47	142	0	54	161	0	5	8	0
	30-<40	24	94	0	166	601	0	145	596	0	174	648	0	42	135	0
	40-<50	58	219	0	326	1482	0	215	1036	0	282	1291	0	56	261	0
	50-<60	68	319	0	274	1087	2	200	817	1	256	1226	3	52	183	0
	60-<70	35	170	0	116	488	1	111	512	1	151	666	1	34	165	0
	70-<80	14	41	0	61	259	1	53	197	2	56	231	1	20	91	0
	Total	115	854	0	584	4019	4	439	3300	4	539	4222	5	124	843	0
Small bowel	25-<30	4	11	0	46	101	0	46	137	0	54	161	0	5	8	0
	30-<40	24	94	0	166	601	0	142	580	1	172	651	0	42	135	0
	40-<50	58	219	0	325	1475	1	212	1014	0	278	1258	3	56	261	0
	50-<60	69	323	0	276	1088	0	193	785	3	251	1199	6	52	183	0
	60-<70	35	170	0	117	490	0	109	503	4	154	674	4	34	165	0
	70-<80	14	41	0	61	257	0	52	192	1	54	221	1	20	91	0
	Total	116	858	0	586	4011	1	429	3212	9	536	4164	14	124	843	0
Urothelial	25-<30	4	11	0	46	101	0	47	142	0	54	161	0	5	8	0
	30-<40	24	94	0	166	601	0	145	596	0	173	651	0	42	135	0
	40-<50	58	219	0	327	1483	0	219	1046	1	278	1260	6	56	261	0
	50-<60	69	323	0	277	1096	2	202	821	4	244	1158	5	52	183	0
	60-<70	35	170	0	115	474	2	112	514	3	137	580	5	33	164	0
	70-<80	14	41	1	59	248	0	53	195	1	50	196	3	18	78	0
	Total	116	858	1	585	4003	4	445	3314	9	524	4006	19	122	829	0
Female breast	25-<30	3	8	0	29	65	0	26	80	0	28	88	0	1	3	0
	30-<40	13	54	0	85	320	0	76	294	0	84	300	0	21	64	0
	40-<50	31	119	0	172	750	2	101	474	1	126	577	1	36	169	2
	50-<60	35	157	1	142	532	0	87	346	2	111	540	3	29	109	0
	60-<70	14	67	0	65	286	0	51	237	0	79	341	2	14	80	1
	70-<80	6	15	0	35	140	1	25	108	0	31	117	0	9	26	1
	Total	58	420	1	307	2093	3	214	1539	3	256	1963	6	60	451	4
Ovarian	25-<30	3	8	0	29	65	0	25	78	0	27	86	0	1	3	0
	30-<40	12	53	0	82	310	0	75	292	0	77	282	1	21	64	0
	40-<50	30	119	0	156	675	0	89	406	0	100	431	2	31	159	0
	50-<60	35	164	0	122	435	0	66	261	0	70	280	1	20	72	0
	60-<70	14	64	0	49	214	0	42	187	0	47	234	1	10	49	0
	70-<80	5	8	0	26	105	0	17	68	0	23	84	0	6	21	0
	Total	56	417	0	270	1805	0	187	1292	0	202	1397	5	48	368	0
Endometrial	25-<30	3	8	0	29	65	0	25	75	0	28	88	0	1	3	0
	30-<40	12	53	0	83	316	0	72	271	0	74	285	1	20	58	0
	40-<50	26	98	0	148	604	3	80	363	6	89	375	5	32	144	1
	50-<60	32	144	0	101	338	2	45	166	1	46	163	6	15	64	0
	60-<70	14	64	0	38	169	0	23	92	1	22	96	1	8	35	0
	70-<80	5	8	0	22	90	0	6	26	1	7	13	0	3	11	0
	Total	53	376	0	254	1581	5	152	994	9	163	1019	13	45	315	1

[#] Without malignant neoplasms of skin except melanoma

* Number of patients who entered the subgroup in the corresponding age group

[$] Person-years

[§] Number of incident cancer events

Table S4: Types of incident cancers considered as "any cancer".

	FCCX	LLS	LS			Total
			MLH1	*MSH2*	*MSH6*	
**Patients with incident cancer, number**	2	8	13	27	3	53
Adrenal gland	0	0	1	0	0	1
Breast	0	0	1	2	0	3
Colorectal	0	5	7	9	1	22
Hepatobiliary	0	0	0	3	0	3
Malignant melanoma of skin	0	0	1	1	0	2
Malignant neoplasms of skin except melanoma	1	0	0	4	0	5
Non-Hodgkin lymphoma	0	1	0	0	0	1
Ovary	0	0	0	2	0	2
Pancreas	1	0	0	0	0	1
Prostate	0	0	0	1	0	1
Small bowel	0	0	0	2	0	2
Urinary tract	0	0	0	1	0	1
Uterus	0	2	3	2	2	9

Table S5: Cumulative cancer risks (%) by age for female patients.

Cancer type	Age (years)	FCCX	LLS	LS	LS		
					MLH1	*MSH2*	*MSH6*
Any	40	0.0 (-)	12.8 (3.3-42.8)	10.9 (3.9-28.3)	12.0 (3.1-40.6)	13.5 (3.1-48.5)	0.0 (-)
	50	14.3 (2.1-66.6)	17.4 (5.9-45.4)	29.7 (18.7-45.1)	27.0 (13.0-50.9)	39.4 (21.6-64.4)	10.0 (1.5-52.7)
	60	14.3 (2.1-66.6)	29.1 (13.0-57.1)	47.6 (33.8-63.8)	43.2 (23.1-70.4)	60.7 (39.7-82.1)	28.0 (7.2-76.2)
	70	31.4 (8.8-78.7)	29.1 (13.0-57.1)	60.1 (42.0-78.7)	-	67.3 (45.7-87.0)	64.0 (21.6-98.6)
Colorectal	40	0.0 (-)	12.6 (3.2-42.3)	6.6 (1.5-25.8)	5.3 (0.8-31.9)	10.0 (1.5-52.7)	0.0 (-)
	50	0.0 (-)	12.6 (3.2-42.3)	10.9 (4.2-26.6)	5.3 (0.8-31.9)	18.9 (6.5-47.9)	0.0 (-)
	60	0.0 (-)	12.6 (3.2-42.3)	18.5 (9.6-33.9)	12.0 (3.1-40.6)	29.2 (14.1-54.4)	0.0 (-)
	70	0.0 (-)	12.6 (3.2-42.3)	38.7 (23.1-60.0)	12.0 (3.1-40.6)	49.6 (29.9-73.4)	25.0 (3.9-87.2)
Stomach	40	0.0 (-)	0.0 (-)	0.0 (-)	0.0 (-)	0.0 (-)	0.0 (-)
	50	0.0 (-)	0.0 (-)	0.0 (-)	0.0 (-)	0.0 (-)	0.0 (-)
	60	0.0 (-)	3.0 (0.4-19.6)	2.1 (0.5-8.2)	0.0 (-)	3.6 (0.9-13.6)	0.0 (-)
	70	0.0 (-)	3.0 (0.4-19.6)	3.2 (1.0-9.8)	2.9 (0.4-19.1)	3.6 (0.9-13.6)	0.0 (-)
Small bowel	40	0.0 (-)	0.0 (-)	1.2 (0.2-8.4)	2.7 (0.4-17.7)	0.0 (-)	0.0 (-)
	50	0.0 (-)	1.3 (0.2-8.6)	1.2 (0.2-8.4)	2.7 (0.4-17.7)	0.0 (-)	0.0 (-)
	60	0.0 (-)	1.3 (0.2-8.6)	4.1 (1.5-10.5)	5.7 (1.5-21.1)	3.6 (0.9-13.8)	0.0 (-)
	70	0.0 (-)	1.3 (0.2-8.6)	10.4 (5.2-20.2)	13.3 (5.0-32.4)	10.0 (3.7-25.3)	0.0 (-)
Urothelial	40	0.0 (-)	0.0 (-)	0.0 (-)	0.0 (-)	0.0 (-)	0.0 (-)
	50	0.0 (-)	0.0 (-)	1.6 (0.4-6.2)	0.0 (-)	3.3 (0.8-12.6)	0.0 (-)
	60	0.0 (-)	3.2 (0.8-12.0)	4.3 (1.8-10.1)	2.5 (0.4-16.5)	6.7 (2.6-16.8)	0.0 (-)
	70	0.0 (-)	6.6 (2.0-20.8)	9.1 (4.4-18.2)	2.5 (0.4-16.5)	15.9 (7.5-31.7)	0.0 (-)

Table S6: Cumulative cancer risks (%) by age for male patients.

					LS		
**Cancer type**	**Age (years)**	**FCCX**	**LLS**	**LS**	***MLH1***	***MSH2***	***MSH6***
Any	40	0.0 (-)	9.1 (1.3-49.2)	14.5 (6.3-31.5)	19.2 (5.0-59.0)	15.0 (5.1-40.1)	0.0 (-)
	50	0.0 (-)	21.3 (7.3-53.3)	19.5 (9.8-36.6)	19.2 (5.0-59.0)	24.0 (10.7-48.5)	0.0 (-)
	60	0.0 (-)	-	51.0 (32.4-72.8)	44.5 (17.3-83.8)	61.4 (37.0-86.0)	0.0 (-)
	70	0.0 (-)	-	65.7 (43.7-86.4)	100.0 (-)	80.7 (47.4-98.5)	0.0 (-)
Colorectal	40	0.0 (-)	9.1 (1.3-49.2)	11.4 (4.4-27.7)	17.9 (4.7-55.6)	9.9 (2.5-35.1)	0.0 (-)
	50	0.0 (-)	21.3 (7.3-53.3)	13.5 (5.8-29.6)	17.9 (4.7-55.6)	13.8 (4.5-37.6)	0.0 (-)
	60	0.0 (-)	30.0 (12.2-62.5)	40.0 (22.7-63.7)	39.4 (15.6-77.2)	46.0 (22.9-76.8)	0.0 (-)
	70	0.0 (-)	-	46.7 (27.6-70.6)	100.0 (-)	46.0 (22.9-76.8)	0.0 (-)
Stomach	40	0.0 (-)	0.0 (-)	0.0 (-)	0.0 (-)	0.0 (-)	0.0 (-)
	50	0.0 (-)	0.0 (-)	0.0 (-)	0.0 (-)	0.0 (-)	0.0 (-)
	60	0.0 (-)	1.6 (0.2-10.6)	1.6 (0.4-6.5)	1.5 (0.2-10.1)	1.8 (0.3-12.2)	0.0 (-)
	70	0.0 (-)	7.7 (1.6-33.4)	2.9 (0.9-9.1)	1.5 (0.2-10.1)	4.4 (1.1-16.8)	0.0 (-)
Small bowel	40	0.0 (-)	0.0 (-)	0.0 (-)	0.0 (-)	0.0 (-)	0.0 (-)
	50	0.0 (-)	0.0 (-)	2.5 (0.8-7.6)	0.0 (-)	4.8 (1.6-14.3)	0.0 (-)
	60	0.0 (-)	0.0 (-)	7.5 (3.9-13.9)	4.0 (1.0-15.1)	10.7 (5.2-21.2)	0.0 (-)
	70	0.0 (-)	0.0 (-)	12.6 (7.4-21.2)	10.1 (3.8-25.5)	16.4 (8.6-30.3)	0.0 (-)
Urothelial	40	0.0 (-)	0.0 (-)	0.0 (-)	0.0 (-)	0.0 (-)	0.0 (-)
	50	0.0 (-)	0.0 (-)	3.8 (1.6-9.0)	1.5 (0.2-10.0)	6.2 (2.3-15.9)	0.0 (-)
	60	0.0 (-)	0.0 (-)	8.6 (4.8-15.2)	8.5 (3.2-21.4)	10.2 (5.0-20.3)	0.0 (-)
	70	0.0 (-)	6.2 (0.9-36.8)	15.8 (9.6-25.2)	18.3 (8.9-35.6)	16.3 (8.4-30.4)	0.0 (-)

Figure S7: Age-dependent cumulative cancer risks by risk group and sex.

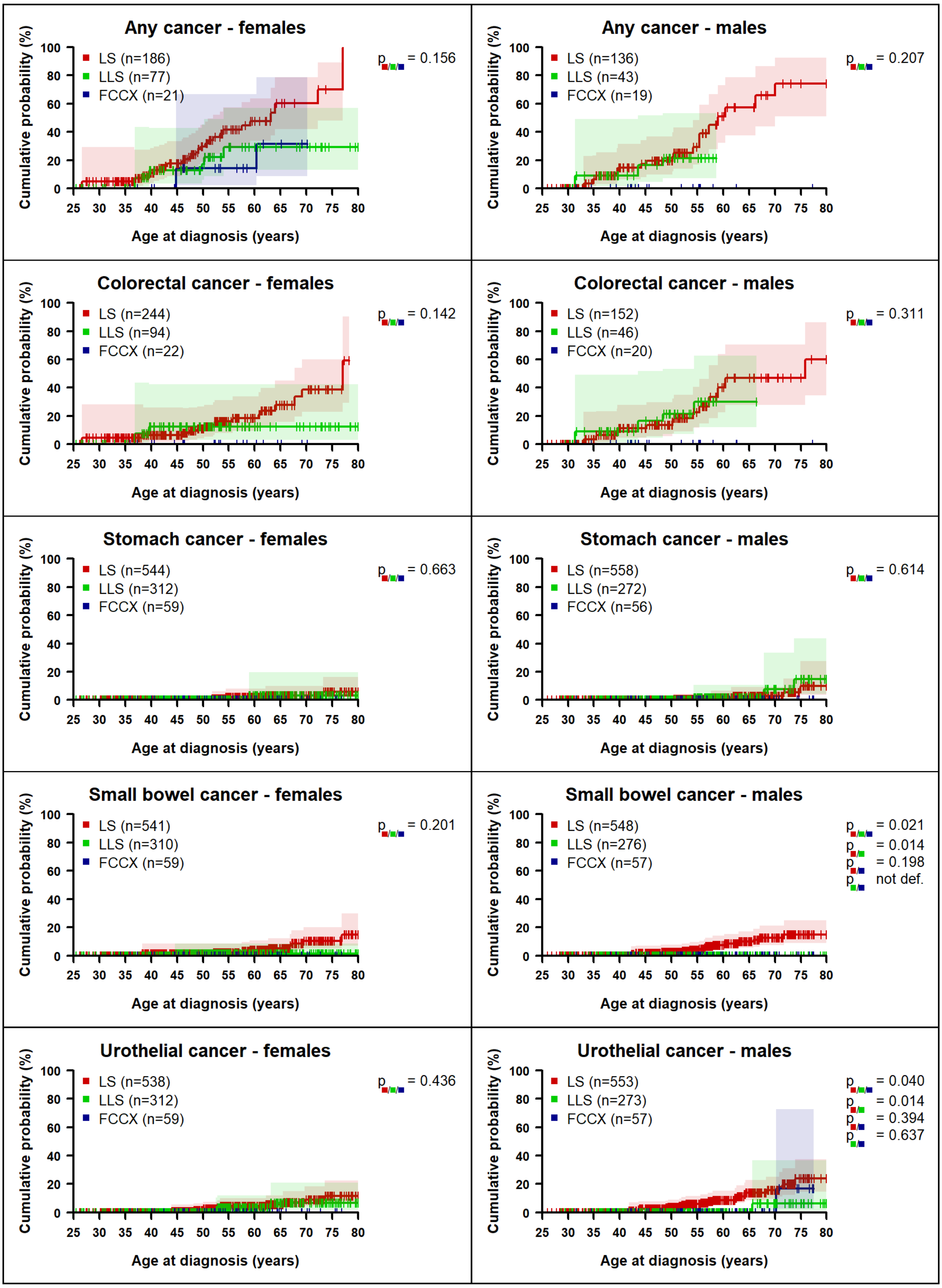

not def. = not definable
Shaded areas indicate 95% confidence intervals.

Figure S8: Age-dependent cumulative cancer risks of LS patients by gene and sex.

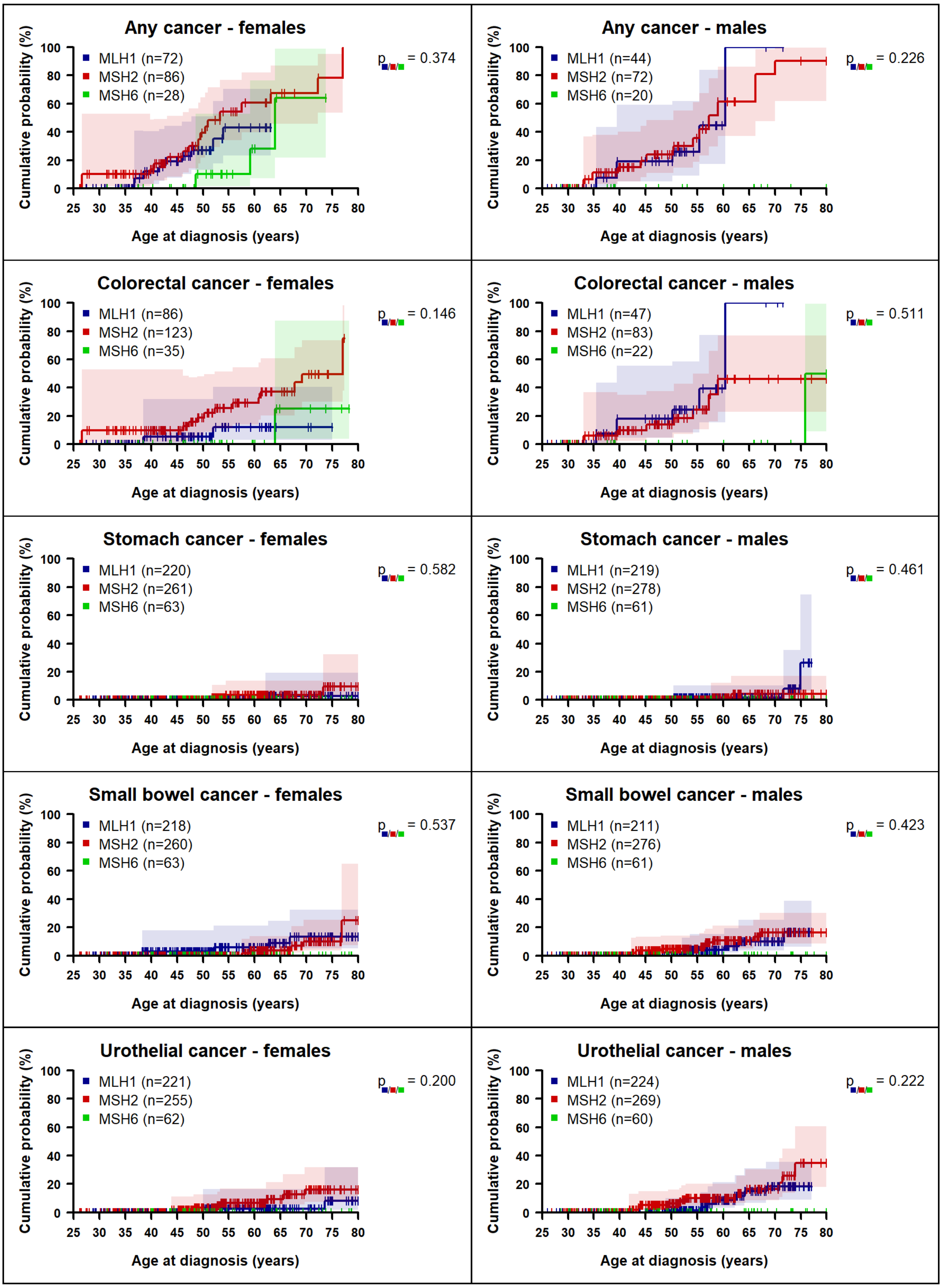

Shaded areas indicate 95% confidence intervals.

Figure S9: Comparison with general population risks: standardised incidence ratios (SIRs) with 95% confidence interval for female patients.

Cancer type	Risk group	SIR (95% CI)	LS by gene	SIR (95% CI)
Any*	LS	5.9 (3.8-8.7)	MLH1	5.6 (2.4-10.9)
	LLS	2.2 (0.7-5.2)	MSH2	7.2 (3.9-12.4)
	FCCX	1.3 (0.0-7.1)	MSH6	3.5 (0.7-10.3)
Colorectal	LS	23.6 (13.2-38.9)	MLH1	13.9 (1.7-50.1)
	LLS	6.3 (0.8-22.7)	MSH2	33.1 (17.1-57.8)
	FCCX	0.0 (0.0-50.7)	MSH6	7.7 (0.2-43.0)
Stomach	LS	7.7 (2.1-19.8)	MLH1	5.1 (0.1-28.3)
	LLS	3.8 (0.1-21.3)	MSH2	11.7 (2.4-34.3)
	FCCX	0.0 (0.0-70.8)	MSH6	0.0 (0.0-57.2)
Small bowel	LS	115.1 (52.6-218.5)	MLH1	136.1 (37.1-348.6)
	LLS	25.5 (0.6-142.1)	MSH2	127.8 (41.5-298.3)
	FCCX	0.0 (0.0-430.0)	MSH6	0.0 (0.0-380.0)
Urothelial	LS	27.7 (12.7-52.6)	MLH1	15.4 (1.9-55.8)
	LLS	18.2 (3.8-53.2)	MSH2	45.7 (18.4-94.1)
	FCCX	0.0 (0.0-111.8)	MSH6	0.0 (0.0-87.7)

(Forest plot axes: 0.01 0.1 1 10 100)

* Without malignant neoplasms of skin except melanoma

Figure S10: Comparison with general population risks: standardised incidence ratios (SIRs) with 95% confidence interval for male patients.

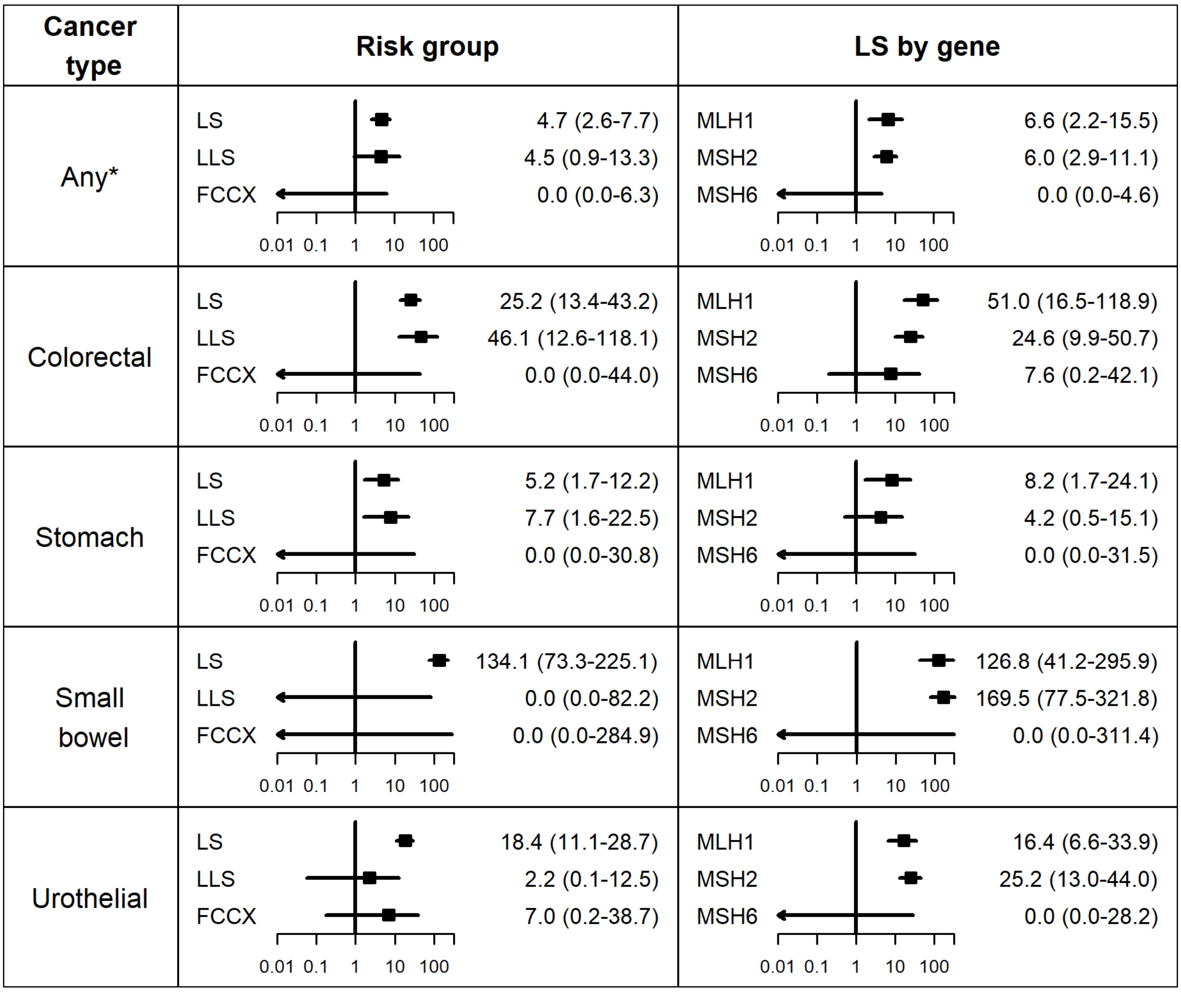

* Without malignant neoplasms of skin except melanoma
